# Supplementary material for: Dissecting the Predictive Accuracy of Polygenic Indexes for Behavioral Phenotypes Across Genetic Ancestries
Source: Res Sq. 2025 Oct 3:rs.3.rs-7584560. Preprint. [Version 1] doi: 10.21203/rs.3.rs-7584560/v1 (PMC12622163; doi:10.21203/rs.3.rs-7584560/v1)
Supplement: Supplement 1 [file NIHPPrs7584560v1-supplement-1.pdf]

5 Supplementary Information

5.1 Estimation of Standard Errors and Confidence Intervals

5.1.1 Incremental R^2 and Observed Relative PGI Predictive Accuracy

We employed a bootstrap procedure with 1,000 replications to estimate the standard errors and construct 95% confidence intervals for incremental R^2 and RA_{Obs}. This method helps to approximate the sampling distribution of these estimators, assuming that our sample adequately represents the population. In each replication, a new sample was drawn with replacement from the original dataset, and R^2 and RA_{Obs} were recalculated using the the analytical models and approaches described in in subsection 2.1. For the construction of 95% confidence intervals, we determined the lower and upper bounds from the 2.5th and 97.5th percentiles of the bootstrap estimates, respectively. This method provides an interval that is expected to contain the true values of R^2 and RA_{Obs} with 95% probability, assuming the bootstrap replicates reflect the true variability of these estimators.

5.1.2 Expected Relative PGI Predictive Accuracy

To estimate the standard errors for the expected relative PGI predictive accuracy when RA_{Expected} is defined as a function of just MAF and LD, we implemented a leave-one-chromosome-out jackknife approach. This method involves sequentially excluding each of the 22 autosomal chromosomes from the dataset and using the remaining chromosomes to infer relevant population parameters from the 1000 Genomes Project Reference Panel, specifically the minor allele frequencies (MAF) for the candidate causal SNPs and PGI-SNPs, and the mean correlations of allele counts between PGI-SNPs and all candidate causal SNPs within a 100 kb window of the top SNPs. These population parameters and the SNP effect size estimates of the PGIs (PGI-SNPs) were then utilized to compute the RA_{Expected} as outlined in Equation 2. After excluding a chromosome and recalculating RA_{Expected} based on the inferred parameters, we assessed the variation in RA_{Expected} across all iterations to compute the standard error:

$$SE_{\text{jackknife}} = \sqrt{\frac{N-1}{N}\sum_{i=1}^{N}(\phi_i - \phi_{\text{mean}})^2} \tag{4}$$

where N represents the total number of chromosomes (22), ϕ_i is the estimate from the i-th iteration with one chromosome omitted, and ϕ_{mean} is the mean of these estimates. This approach provides a robust measure of the standard error by reflecting the stability of RA_{Expected} against the exclusion of any single chromosome's data.

When RA_{Expected} is modeled as a function of MAF, LD and h^2, it is calculated by multiplying Equation 2 with the ratio of the SNP-based heritability of the target (non-EUR) to the reference (EUR) genetic ancestries. The standard error of RA_{Expected} that accounts for all the three factors is estimated by propagating the errors associated with estimating the respective SNP-based heritabilities using the delta-method[48].

5.1.3 Factors Explaining Loss of PGI Predictive Accuracy

To estimate the loss in predictive accuracy of PGIs attributable to MAF and LD, we calculate $LoA_{(\text{LD+MAF})}$ as outlined in Equation (3). We applied the delta method[48] to estimate the variability in $LoA_{(\text{LD+MAF})}$. This method uses a Taylor series expansion to approximate the variance of functions of random variables. Here, the variance of the loss estimate is influenced by the partial derivatives with respect to RA_{Expected} and RA_{Obs}, calculated as follows:

$$\frac{\partial LoA_{(\text{LD+MAF})}}{\partial RA_{\text{Expected}}} = -\frac{100}{1 - RA_{\text{Obs}}}$$
$$\frac{\partial LoA_{(\text{LD+MAF})}}{\partial RA_{\text{Obs}}} = \frac{100 \times (RA_{\text{Expected}} - 1)}{(1 - RA_{\text{Obs}})^2}$$

These derivatives and the observed variances of RA_{pred} and RA_{obs} are then used to estimate the variance of $LoA_{(\text{LD+MAF})}$ as follows:

$$\mathrm{Var}(LoA_{(\mathrm{LD+MAF})}) \approx \left(\frac{\partial LoA_{(\mathrm{LD+MAF})}}{\partial RA_{\mathrm{Expected}}}\right)^2 \mathrm{Var}(RA_{\mathrm{Expected}}) + \left(\frac{\partial LoA_{(\mathrm{LD+MAF})}}{\partial RA_{\mathrm{Obs}}}\right)^2 \mathrm{Var}(RA_{\mathrm{Obs}}) \quad (5)$$

From the variance, the standard error of $LoA_{(\mathrm{LD+MAF})}$ is calculated as:

$$SE_{LoA_{(\mathrm{LD+MAF})}} = \sqrt{\mathrm{Var}(LoA_{(\mathrm{LD+MAF})})} \quad (6)$$

The delta method relies on the assumption that RA_{Expected} and RA_{Obs} are statistically independent. This assumption is justified in our analysis as these parameters are estimated from non-overlapping samples. This approach allows us to effectively quantify the uncertainty surrounding our estimates of $LoA_{(\mathrm{LD+MAF})}$.

5.2 Statistical tests

5.2.1 Testing differences in RA_{Obs} between fGWAS and standard PGIs

For each ancestry–phenotype, we tested the null hypothesis that the mean difference in observed relative accuracy between fGWAS and standard PGIs equals zero (i.e., $RA_{\mathrm{Obs,\ fGWAS}} - RA_{\mathrm{Obs,\ stdGWAS}} = 0$). We used a *paired* nonparametric bootstrap for inference: within each bootstrap replicate, we recomputed RA_{Obs} for fGWAS and standard PGIs on the same resampled data and took their difference, thereby constructing an empirical distribution of the difference without imposing a parametric null[?] . We chose a nonparametric approach because RA_{Obs} is a ratio measure whose sampling distribution can be skewed, heavy-tailed, and heteroskedastic (e.g., when denominators are small or estimates are near zero), making normality-based approximations unreliable. The paired bootstrap preserves the dependence between fGWAS and standard estimates within each resample and naturally accommodates asymmetry. Two-sided 95% confidence intervals for the difference were obtained via the percentile method (2.5th and 97.5th percentiles of the bootstrap distribution). We also reported empirical, two-sided p-values from the same distribution and controlled the false discovery rate across ancestry–phenotype tests using the Benjamini–Hochberg procedure[42]. A difference was deemed statistically significant when its 95% confidence interval excluded zero and the FDR-adjusted p-value met the stated threshold.

5.2.2 Testing differences in $LoA_{(\mathrm{LD+MAF})}$ between UKB and HRS in AFR

To test whether the contribution of LD and MAF to the loss of PGI predictive accuracy differs between UKB and HRS in the AFR genetic ancestry, we compared $LoA_{(\mathrm{LD+MAF})}$ estimates for each phenotype using a two-sided Z-test on the log-transformed values. The log transformation was used to stabilize variance and facilitate interpretation on the ratio scale.
For each cohort and phenotype, we computed the standard error of the log-transformed estimate using the delta method:

$$\mathrm{SE}\big[\log(\widehat{\mathrm{LoA}})\big] = \frac{\mathrm{SE}(\widehat{\mathrm{LoA}})}{\widehat{\mathrm{LoA}}}.$$

Given that the two cohorts are independent, the standard error of the difference in log-transformed values was:

$$\mathrm{SE}_{\log\text{-diff}} = \sqrt{\mathrm{SE}^2_{\log(\mathrm{UKB})} + \mathrm{SE}^2_{\log(\mathrm{HRS})}}.$$

The resulting Z-statistic was:

$$Z = \frac{\log(\widehat{\mathrm{LoA}}_{\mathrm{UKB}}) - \log(\widehat{\mathrm{LoA}}_{\mathrm{HRS}})}{\mathrm{SE}_{\log\text{-diff}}}.$$

Two-sided p-values were obtained from the standard normal distribution, and adjusted for multiple testing using the Benjamini–Hochberg procedure[42]. A phenotype was considered to show a statistically significant difference in $LoA_{(\mathrm{LD+MAF})}$ between cohorts if the FDR-adjusted p-value was below 0.05.

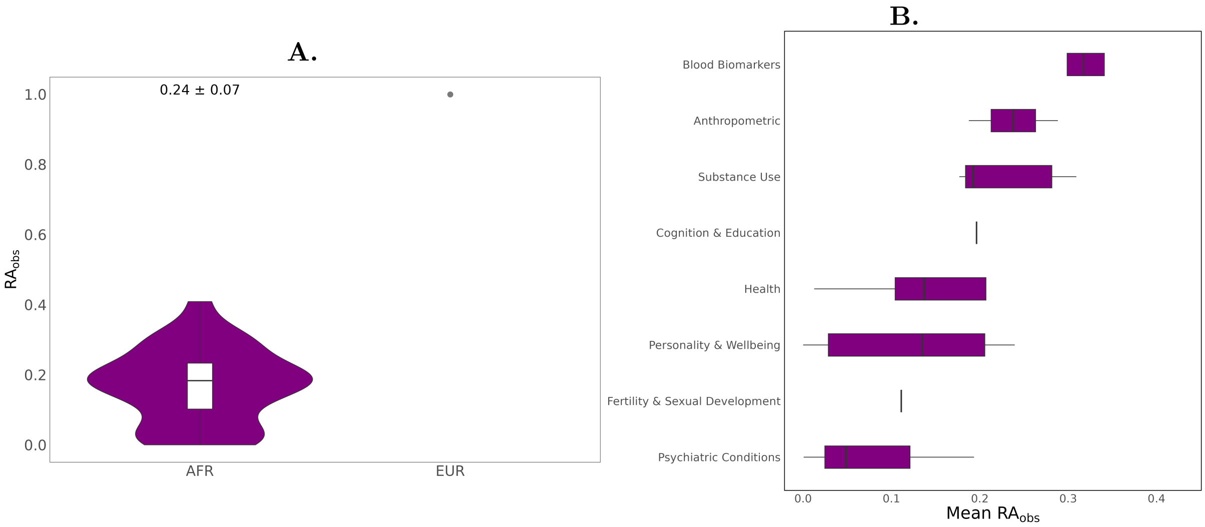

Fig. S1: **Observed relative predictive accuracy of PGIs in African genetic-ancestry samples from the HRS cohort.** **(A)** Violin plot illustrating the distribution of observed relative predictive accuracy (RA_{Obs}) for PGIs across all phenotypes, with an embedded boxplot representing the quartiles. A grey dotted line at $RA_{\text{Obs}} = 1$ represents the EUR genetic-ancestry reference group. The average RA_{Obs} and standard error, estimated via a leave-one-phenotype-out jackknife approach, are displayed above the violin plot. **(B)** Breakdown of RA_{Obs} by eight phenotype categories. Phenotype exclusions, as described in Figure S2, apply here as well, omitting asthma, breast cancer, prostate cancer, bipolar disorder, and schizophrenia due to case proportions below 1%. Moreover, the phenotype alcohol misuse was also removed from the plots as the estimate is too imprecise, distorting the plot (however, all RA_{Obs} are reported in the supplementary tables).

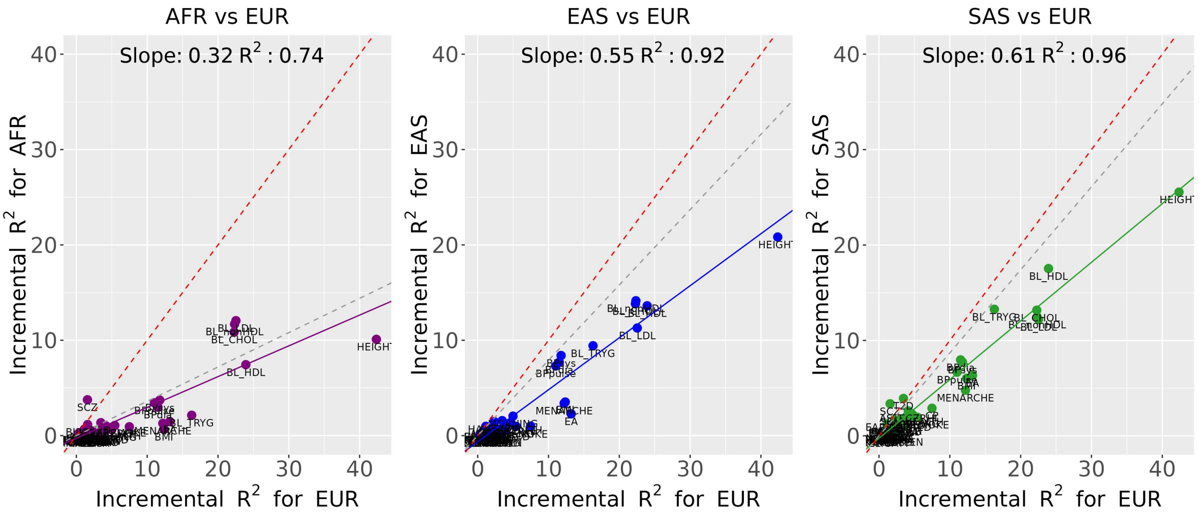

Fig. S2: **Predictive accuracy of polygenic indexes (PGIs) in non-European versus European genetic-ancestry samples in the UKB cohort.** Each panel shows a scatter plot of incremental R^2 values for PGIs in one of three non-European ancestries (AFR: African, EAS: East Asian, SAS: South Asian) compared to EUR (European) incremental R^2 values across various phenotypes. The x-axis in each panel represents the incremental R^2 of a phenotype in EUR, while the y-axis represents the incremental R^2 of the corresponding phenotype for a given non-EUR ancestry. The first panel shows AFR vs. EUR, the second shows EAS vs. EUR, and the third shows SAS vs. EUR. Only phenotypes with an expected $R^2 \geq 1\%$ in EUR ancestry were included, and binary phenotypes with case proportions of less than 1% in the UKB cohort (partition 3) sample—specifically inflammatory bowel disease (IBD), anorexia nervosa, attention deficit and hyperactivity disorder (ADHD), and autism spectrum disorder—were excluded. Points represent individual phenotypes, with colors corresponding to ancestry pairs (AFR: purple, EAS: blue, SAS: green). The solid lines, colored to match each non-EUR ancestry, indicate the fitted linear regression line for each ancestry pair, with the slope and R^2 of this line displayed in the upper right of each panel. The red dashed line represents a slope of 1 (indicating equal predictive accuracy across ancestries), while the secondary gray dashed line denotes the average expected predictive accuracy of PGIs i.e. (RA_{Expected}) that accounts only for minor allele frequency (MAF) and linkage disequilibrium (LD) differences in each non-EUR genetic ancestry (AFR: 0.36, EAS: 0.79, SAS: 0.88).

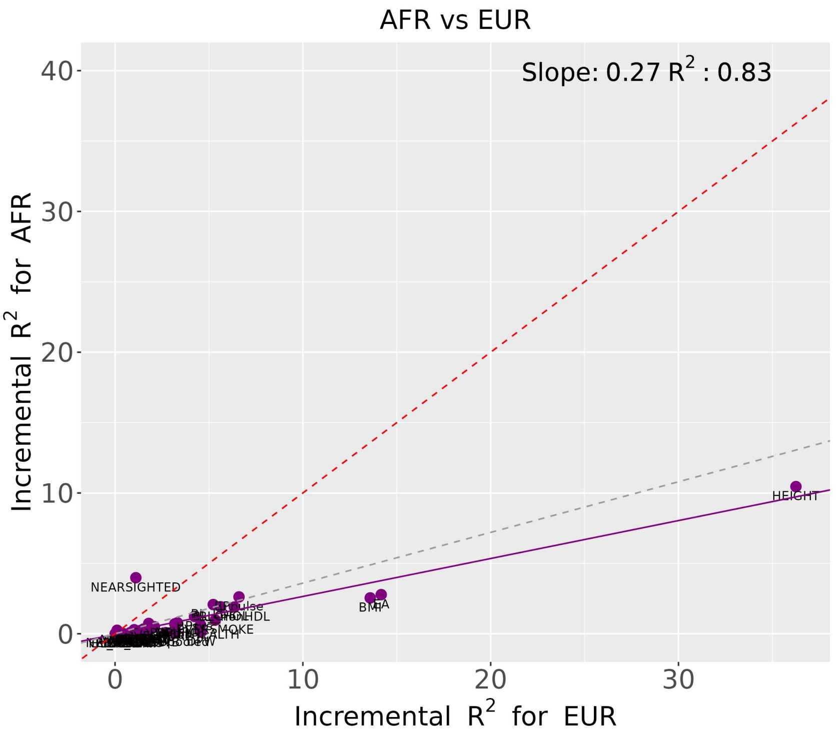

Fig. S3: **Predictive accuracy of polygenic indexes (PGIs) in African versus European genetic-ancestry samples in the HRS cohort.** This panel compares incremental R^2 values for PGIs in AFR (African) versus EUR (European) genetic ancestries across various phenotypes in the HRS cohort. Details follow those described in Figure S1, with binary phenotypes excluded if their case proportions were below 1% in HRS (asthma, breast cancer, prostate cancer, bipolar disorder, and schizophrenia). Points represent individual phenotypes, with a solid regression line fitted for AFR and reference lines indicating the expected predictive accuracy (RA_{Expected}) based on MAF and LD differences.

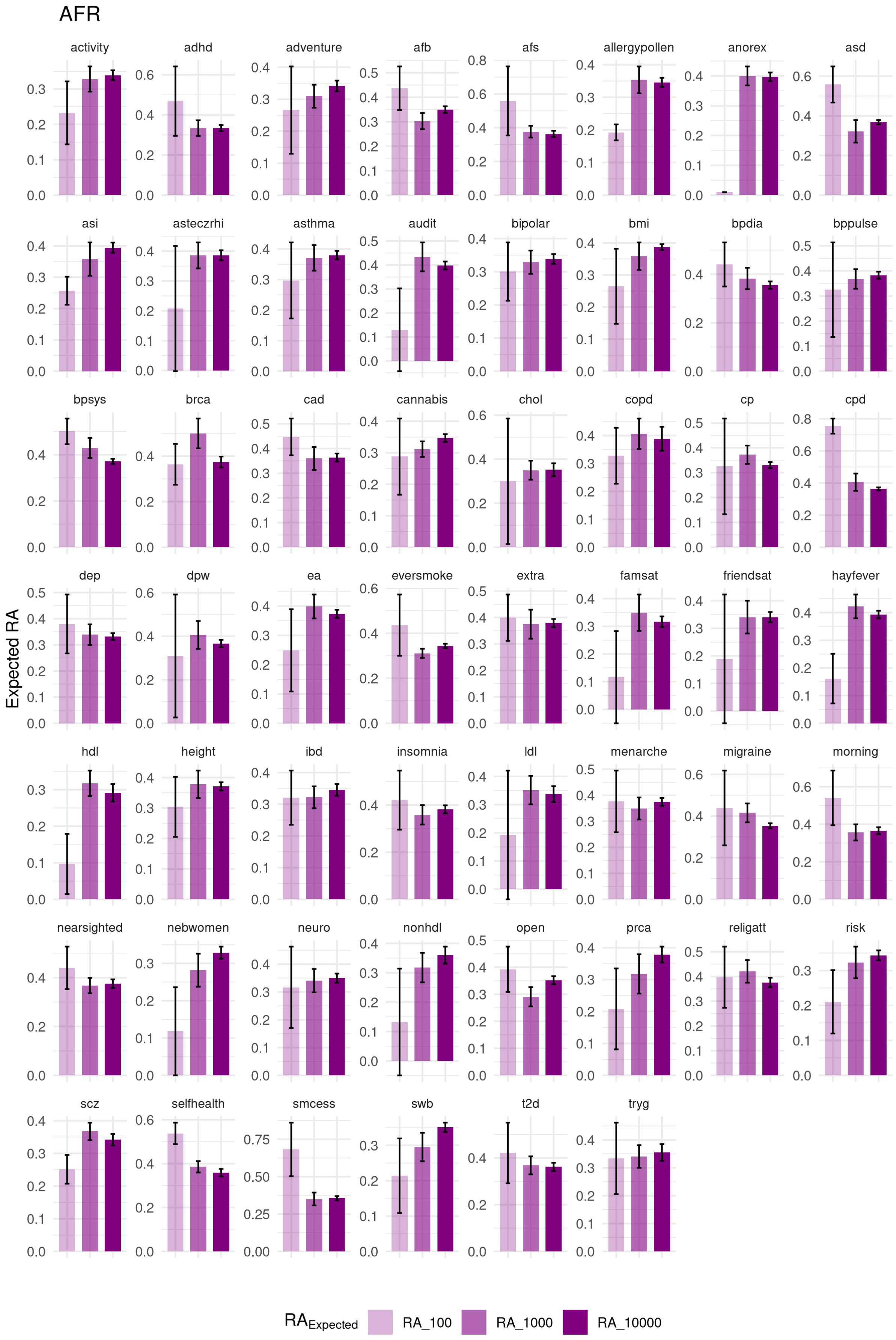

Fig. S4: Expected relative accuracy of PGIs in AFR genetic ancestry by top SNP sets in the UKB cohort

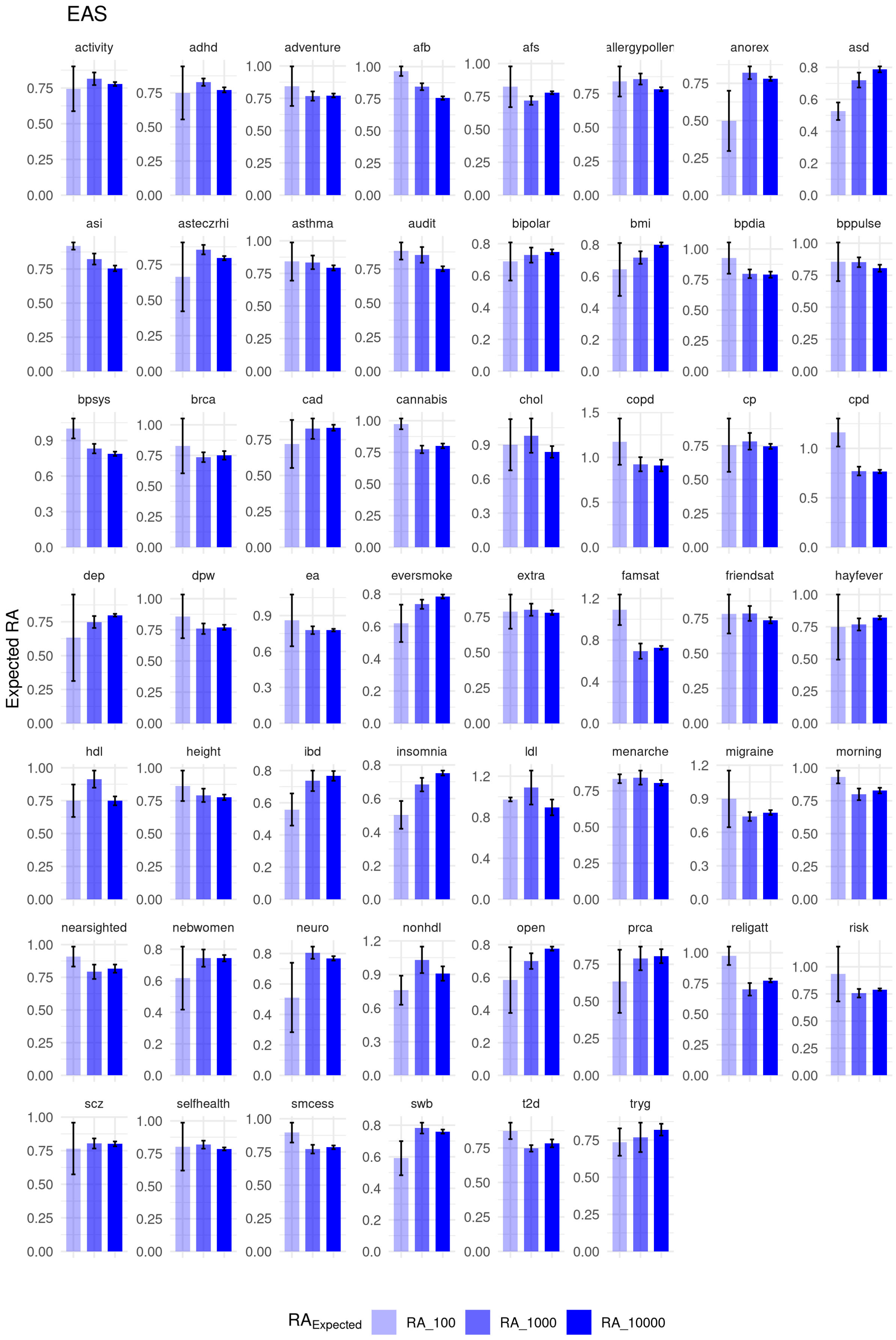

Fig. S5: Expected relative accuracy of PGIs in EAS genetic ancestry by top SNP sets in the UKB cohort

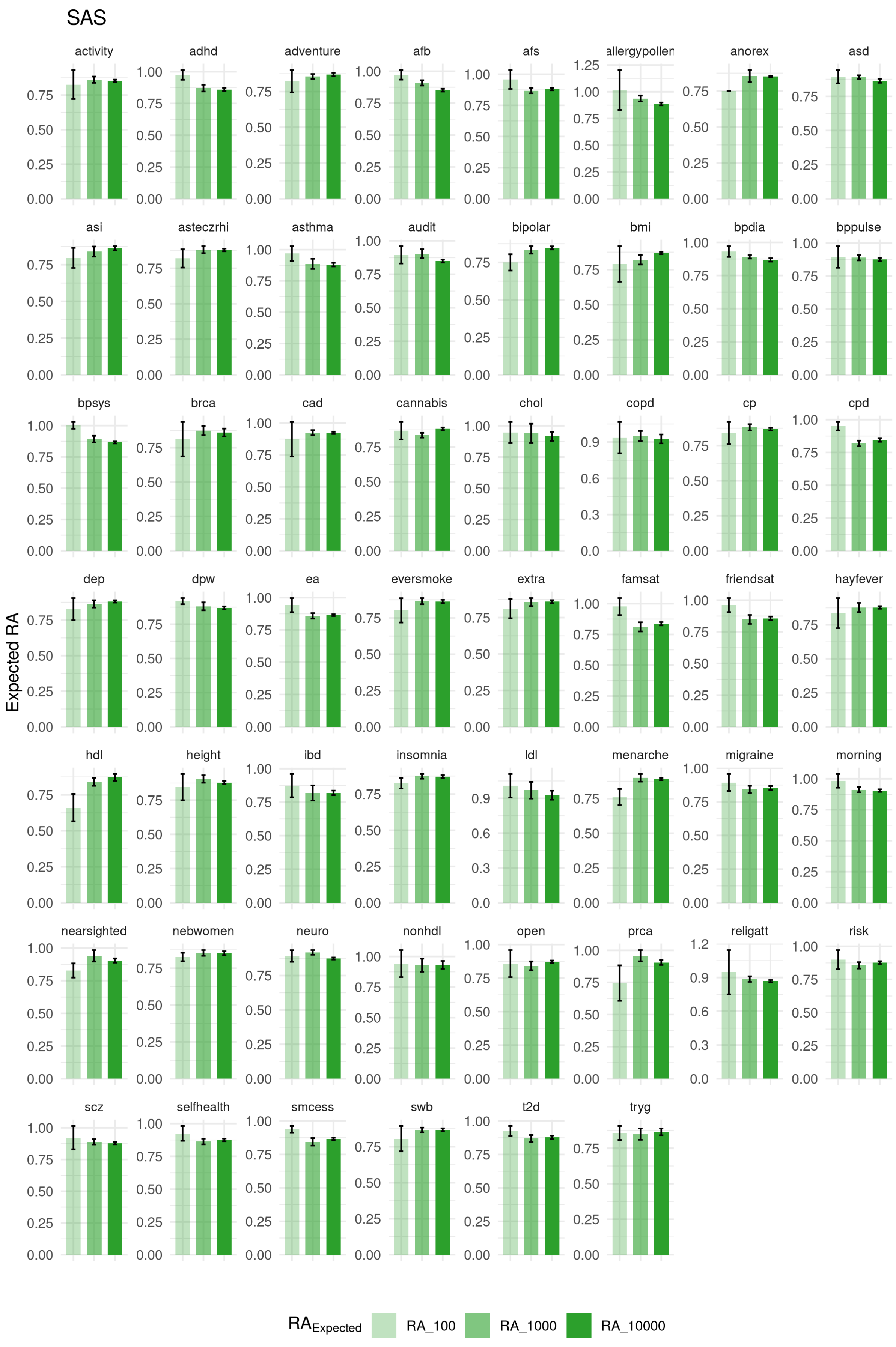

Fig. S6: Expected relative accuracy of PGIs in SAS genetic ancestry by top SNP sets in the UKB cohort

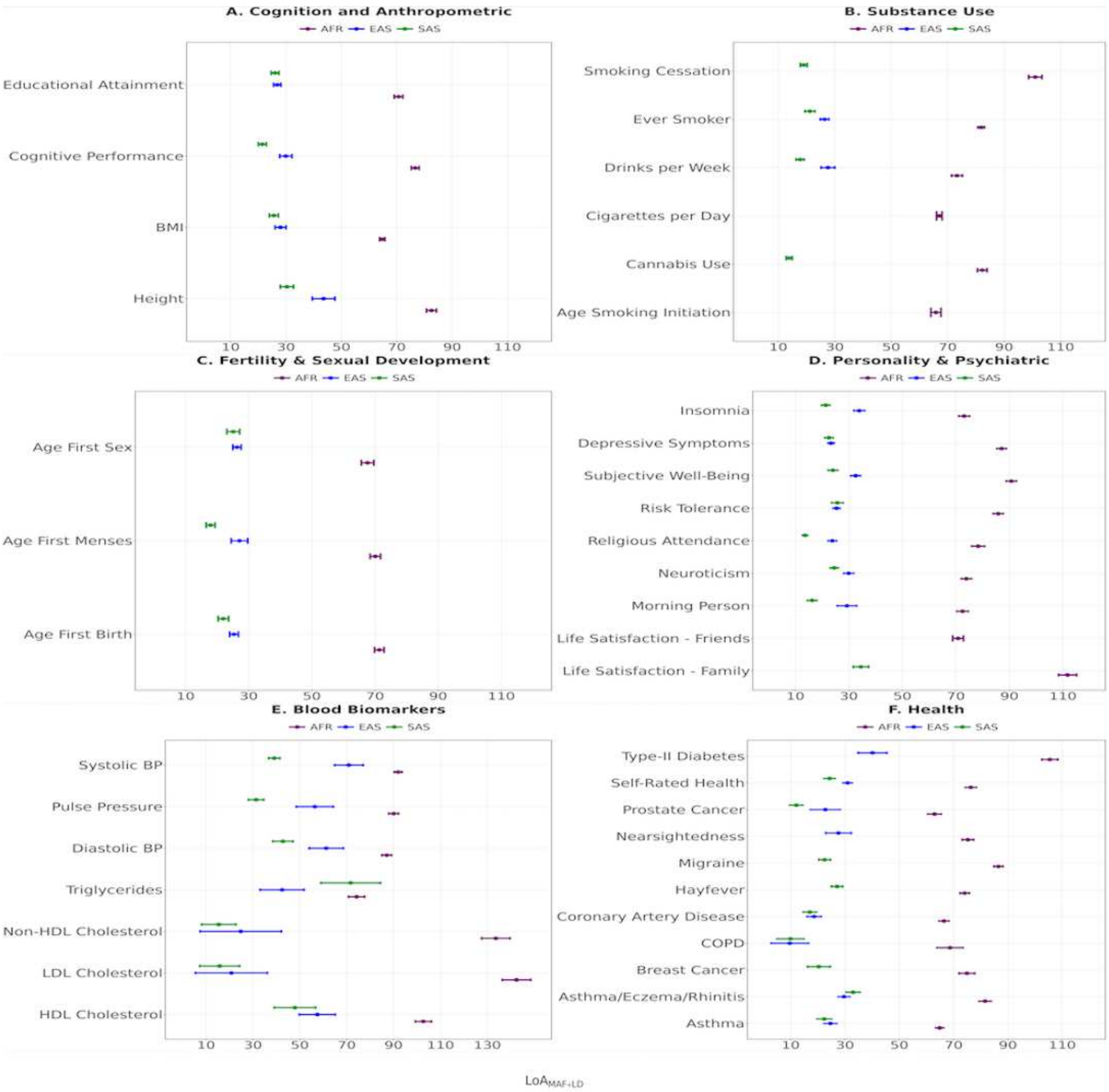

Fig. S7: **Contribution of Minor Allele Frequency (MAF) and Linkage Disequilibrium (LD) to the Loss of PGI Predictive Accuracy by Genetic Ancestry and Phenotype Category in the UKB Cohort.**
This figure presents the contribution of MAF and LD to the loss of PGI predictive accuracy ($LoA_{(\mathrm{LD+MAF})}$) across six phenotype categories in the UKB cohort: cognition and anthropometric traits (Panel A), substance use (Panel B), fertility and sexual development (Panel C), personality and psychiatric traits (Panel D), blood biomarkers (Panel E), and health (Panel F). For each category, $LoA_{(\mathrm{LD+MAF})}$ is shown as point estimates with 95% confidence-interval error bars for the three non-European ancestries—South Asian (SAS), East Asian (EAS), and African (AFR). The x-axis indicates $LoA_{(\mathrm{LD+MAF})}$, and the y-axis lists the phenotypes within each category. Standard errors for $LoA_{(\mathrm{LD+MAF})}$ were computed via the delta method, accounting for variance in both RA_{Expected} and RA_{Obs} in Equation (3) (see Supplementary Note 5.1.3). Binary phenotypes excluded due to low case proportions ($< 1\%$) in UKB are inflammatory bowel disease (IBD), anorexia nervosa, attention-deficit/hyperactivity disorder (ADHD), and autism spectrum disorder (ASD).

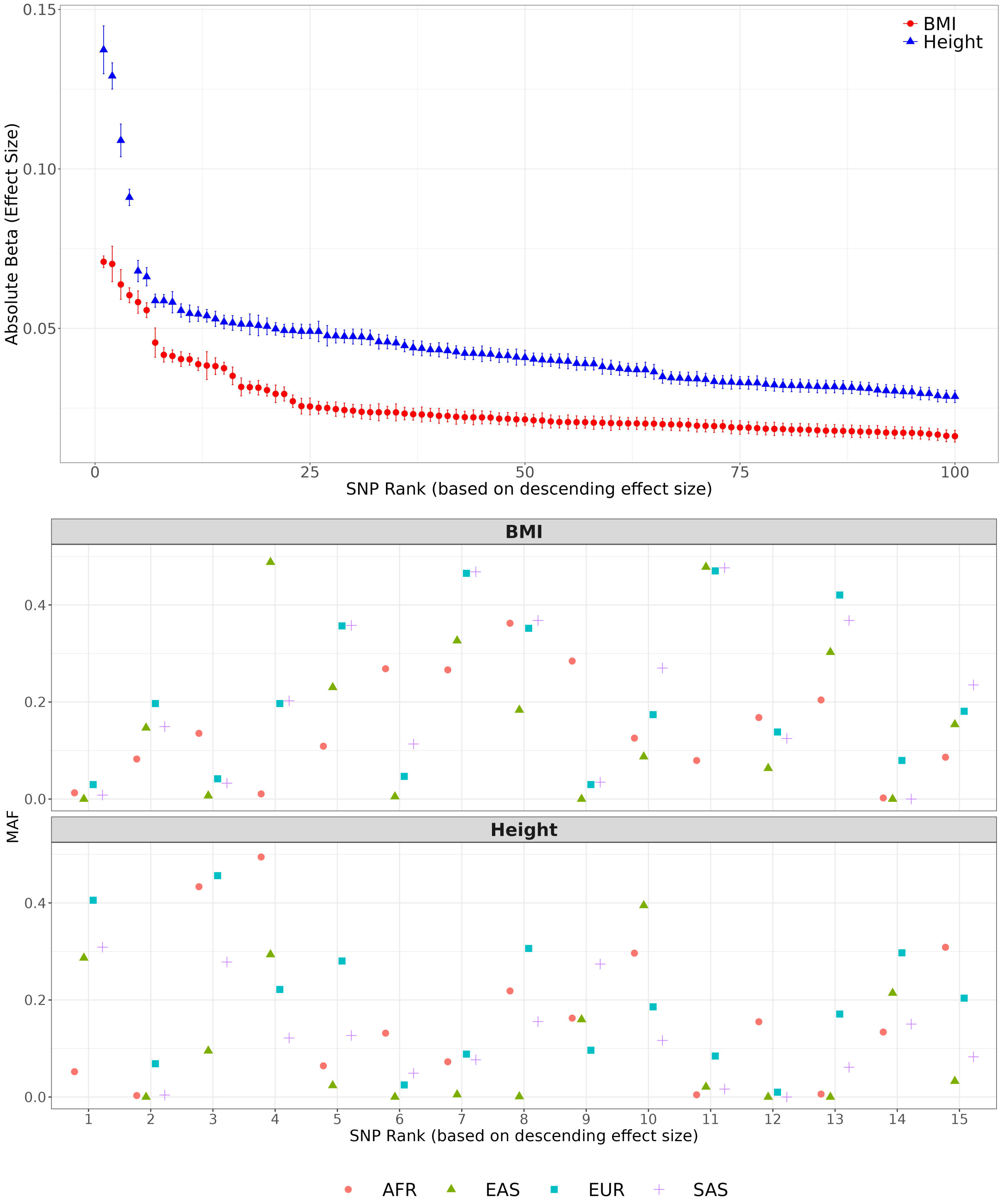

Fig. S8: Effect sizes and minor allele frequencies of SNPs associated with anthropometric traits from standard GWAS. The top 100 SNPs were selected based on their p-values and are ordered by descending absolute effect size. **Top panel:** Absolute effect sizes of these top 100 SNPs, ranked accordingly. **Bottom panel:** Minor allele frequencies (MAF) for the top 15 large-effect SNPs disaggregated by genetic ancestry (EUR, SAS, AFR, and EAS) as estimated from the 1000 Genomes Project (1KGP) reference panel[38]. MAF values were computed as the minimum of the reported alternate allele frequency and its complement (i.e. $\text{MAF} = \min(\text{ALT\_FREQ}, 1 - \text{ALT\_FREQ})$).

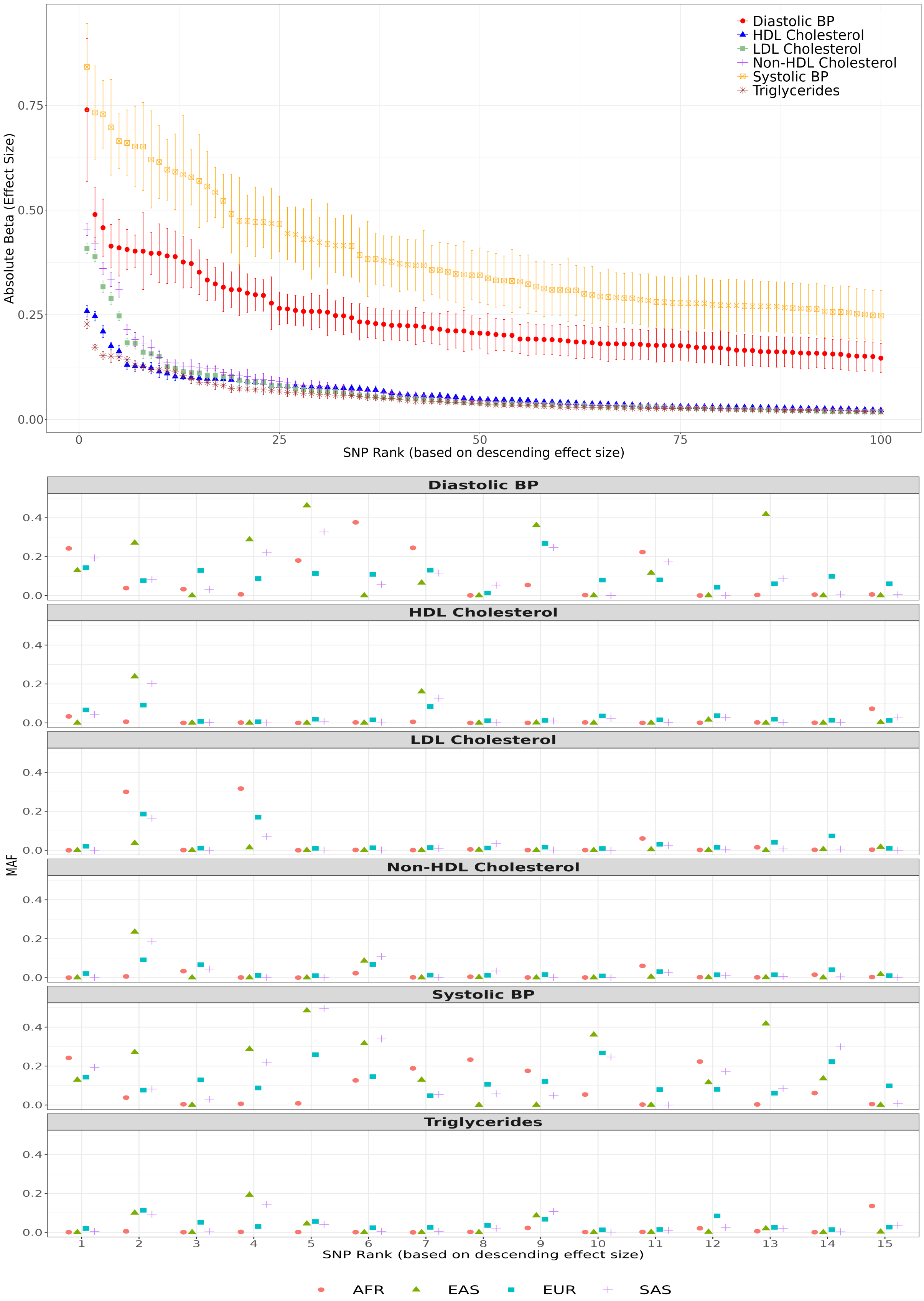

Fig. S9: Effect sizes and minor allele frequencies of SNPs associated with blood biomarker traits from standard GWAS. The top 100 SNPs were selected based on their p-values and are ordered by descending absolute effect size. **Top panel:** Absolute effect sizes of these top 100 SNPs, ranked accordingly. **Bottom panel:** Minor allele frequencies (MAF) for the top 15 large-effect SNPs disaggregated by genetic ancestry (EUR, SAS, AFR, and EAS) as estimated from the 1KGP reference panel[38]. For both panels, MAF is computed as the minimum of the reported alternate allele frequency and its complement (i.e. $\text{MAF} = \min(\text{ALT\_FREQ}, 1 - \text{ALT\_FREQ})$).

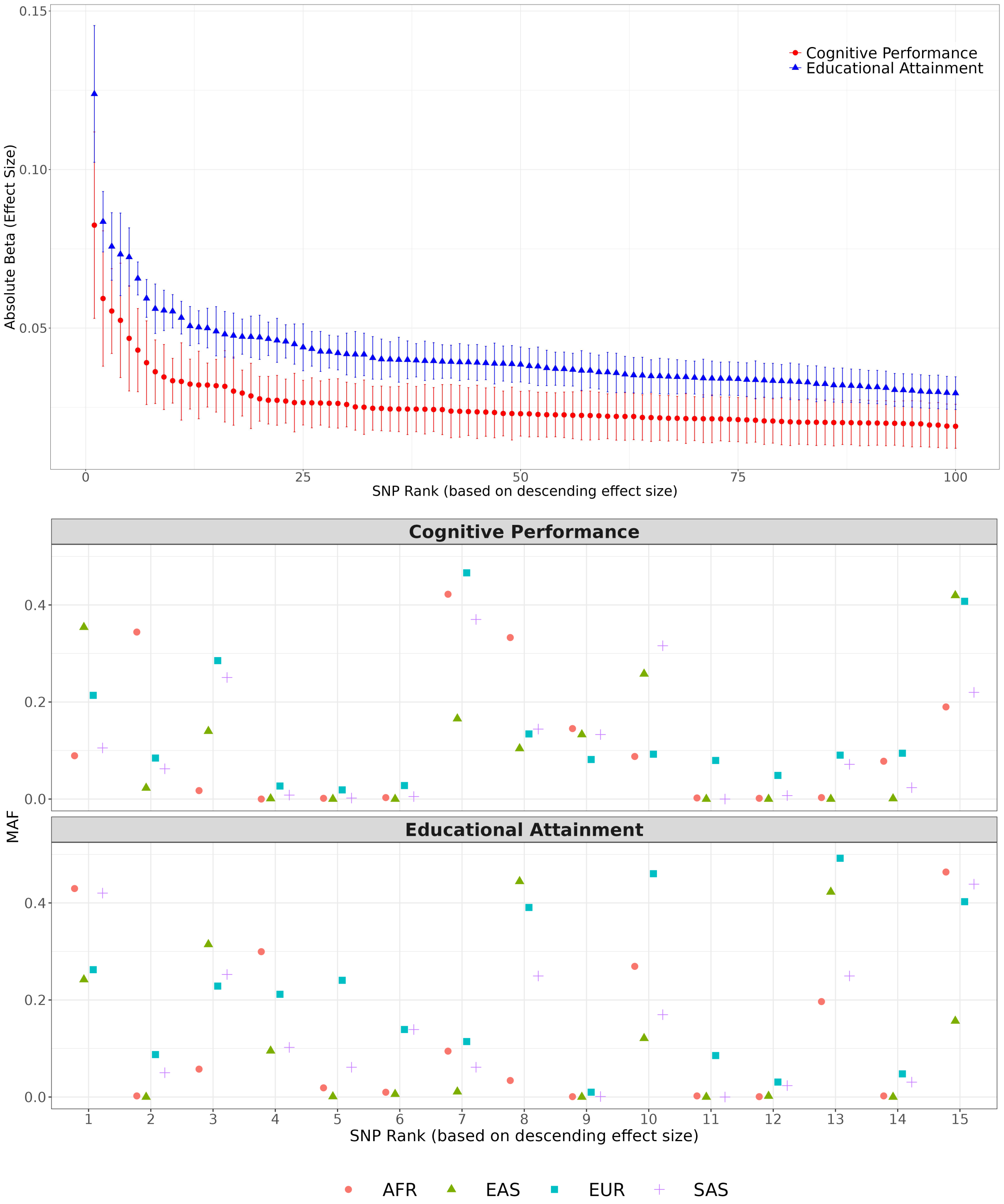

Fig. S10: Effect sizes and minor allele frequencies of SNPs associated with cognition and education traits. The top 100 SNPs were selected based on their p-values and are ordered by descending absolute effect size. **Top panel:** Absolute effect sizes of these top 100 SNPs, ranked accordingly. **Bottom panel:** Minor allele frequencies (MAF) for the top 15 large-effect SNPs disaggregated by genetic ancestry (EUR, SAS, AFR, and EAS) as estimated from the 1KGP reference panel[38]. For both panels, MAF is computed as the minimum of the reported alternate allele frequency and its complement (i.e. $\mathrm{MAF} = \min(\mathrm{ALT\_FREQ}, 1 - \mathrm{ALT\_FREQ})$).

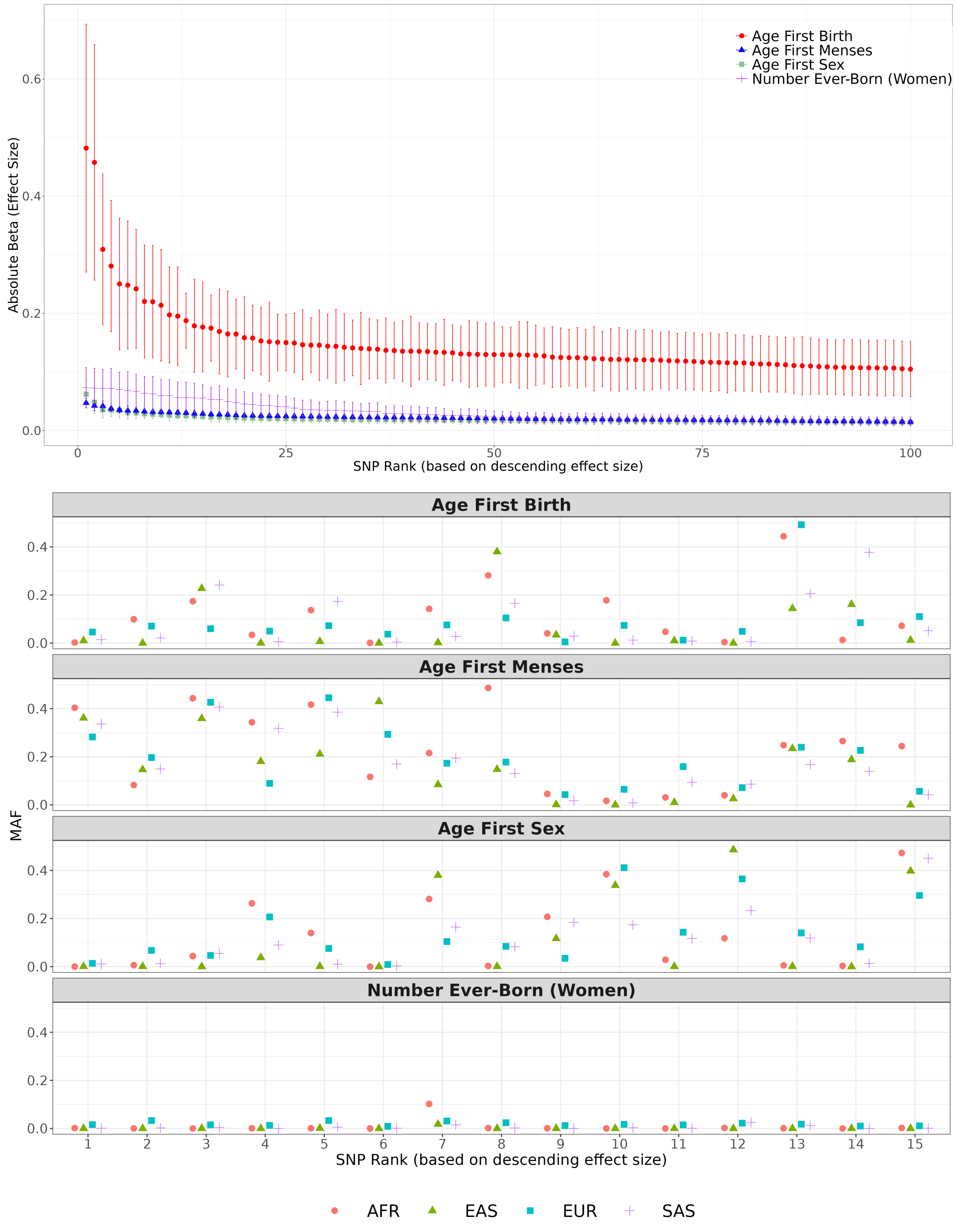

Fig. S11: Effect sizes and minor allele frequencies of SNPs associated with fertility and sexual development traits. The top 100 SNPs were selected based on their p-values and are ordered by descending absolute effect size. **Top panel:** Absolute effect sizes of these top 100 SNPs, ranked accordingly. **Bottom panel:** Minor allele frequencies (MAF) for the top 15 large-effect SNPs disaggregated by genetic ancestry (EUR, SAS, AFR, and EAS) as estimated from the 1KGP reference panel[38]. For both panels, MAF is computed as the minimum of the reported alternate allele frequency and its complement (i.e. $\text{MAF} = \min(\text{ALT\_FREQ}, 1 - \text{ALT\_FREQ})$).

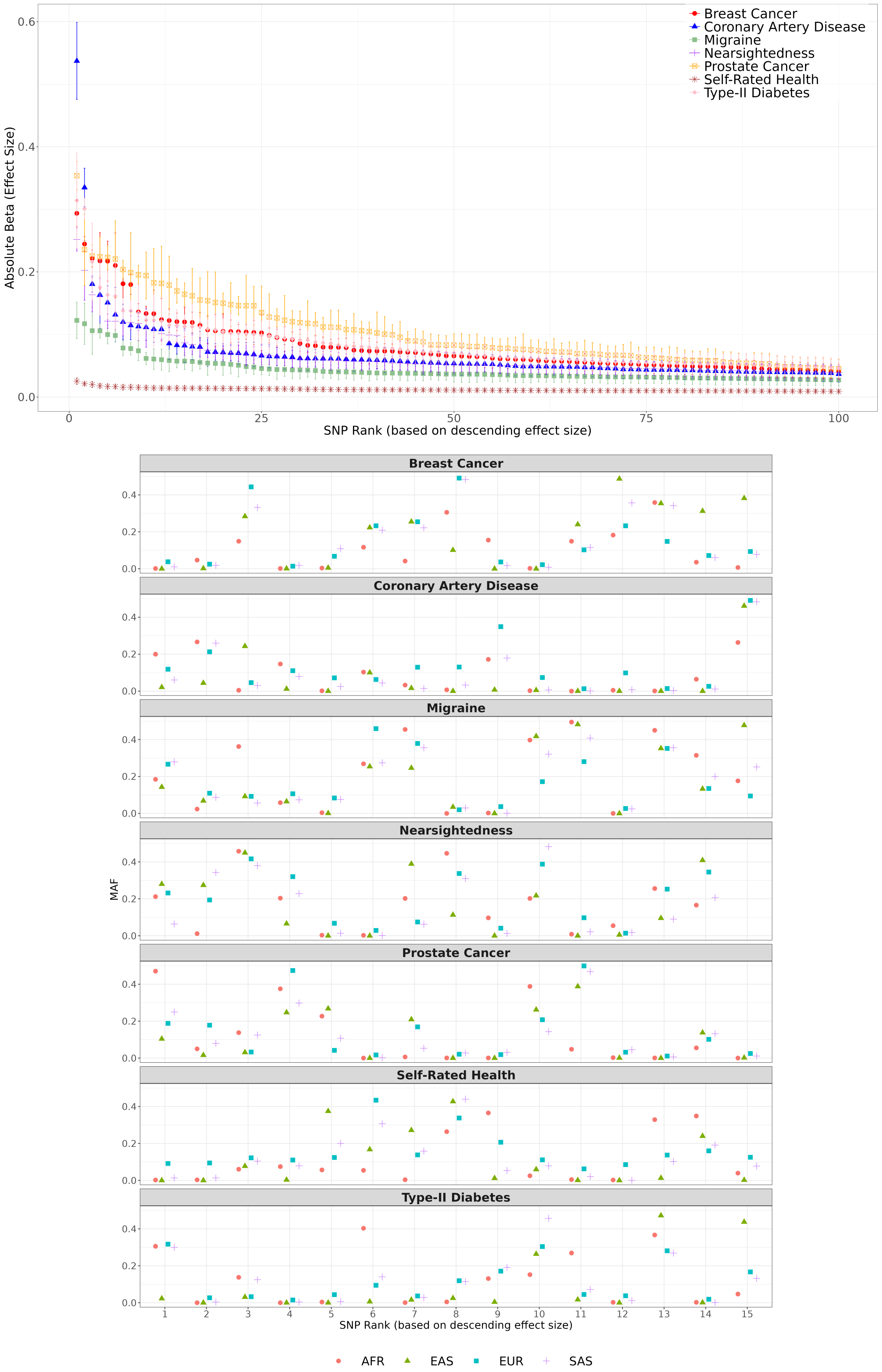

Fig. S12: Effect sizes and minor allele frequencies of SNPs associated with health-related traits (other than respiratory and allergic conditions). The top 100 SNPs were selected based on their p-values and are ordered by descending absolute effect size. **Top panel:** Absolute effect sizes of these top 100 SNPs, ranked accordingly. **Bottom panel:** Minor allele frequencies (MAF) for the top 15 large-effect SNPs disaggregated by genetic ancestry (EUR, SAS, AFR, and EAS) as estimated from the 1KGP reference panel[38]. For both panels, MAF is computed as the minimum of the reported alternate allele frequency and its complement (i.e. $\mathrm{MAF} = \min(\mathrm{ALT\_FREQ}, 1 - \mathrm{ALT\_FREQ})$).

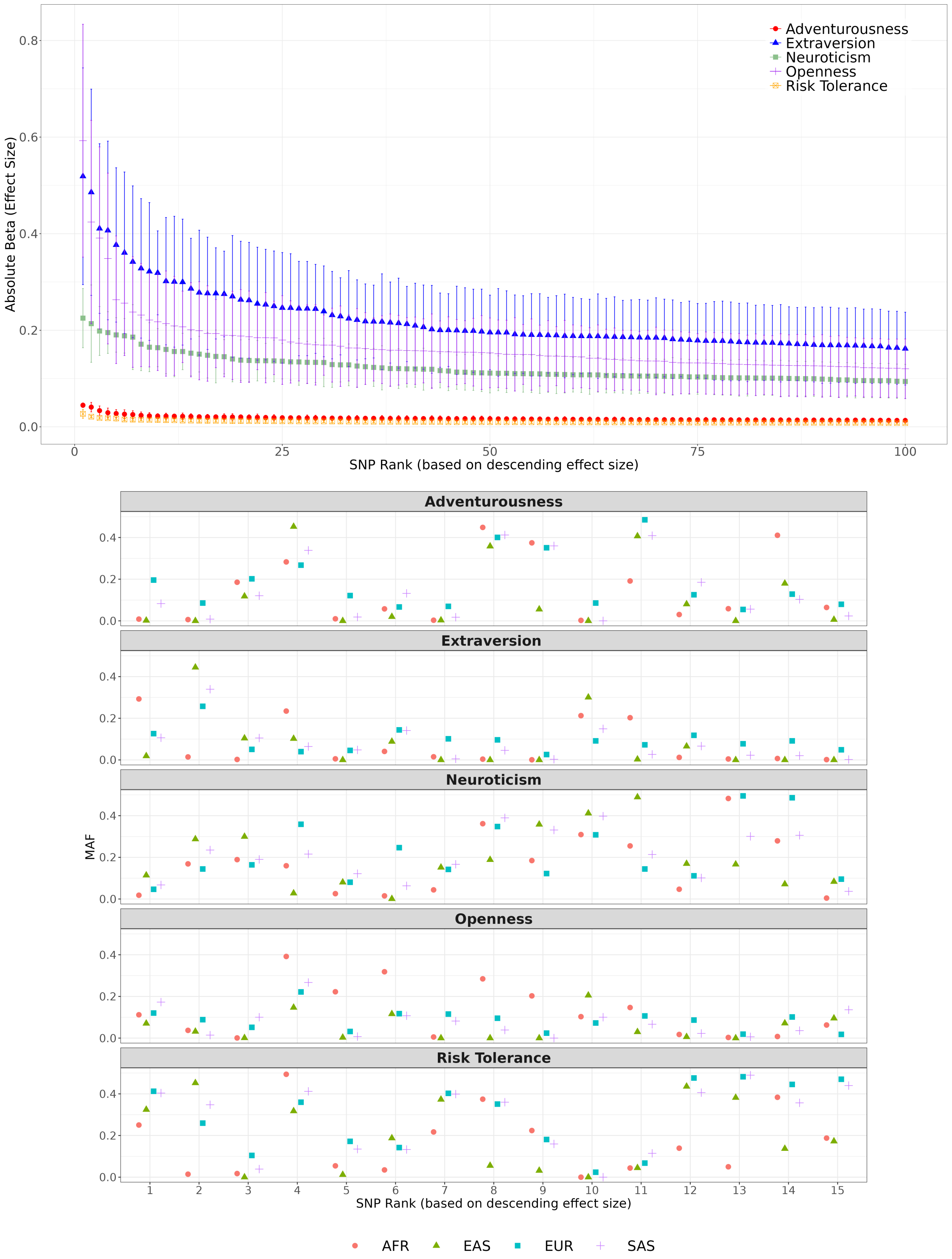

Fig. S13: Effect sizes and minor allele frequencies of SNPs associated with personality traits. The top 100 SNPs were selected based on their p-values and are ordered by descending absolute effect size. **Top panel:** Absolute effect sizes of these top 100 SNPs, ranked accordingly. **Bottom panel:** Minor allele frequencies (MAF) for the top 15 large-effect SNPs disaggregated by genetic ancestry (EUR, SAS, AFR, and EAS) as estimated from the 1KGP reference panel[38]. For both panels, MAF is computed as the minimum of the reported alternate allele frequency and its complement (i.e. $\text{MAF} = \min(\text{ALT\_FREQ}, 1 - \text{ALT\_FREQ})$).

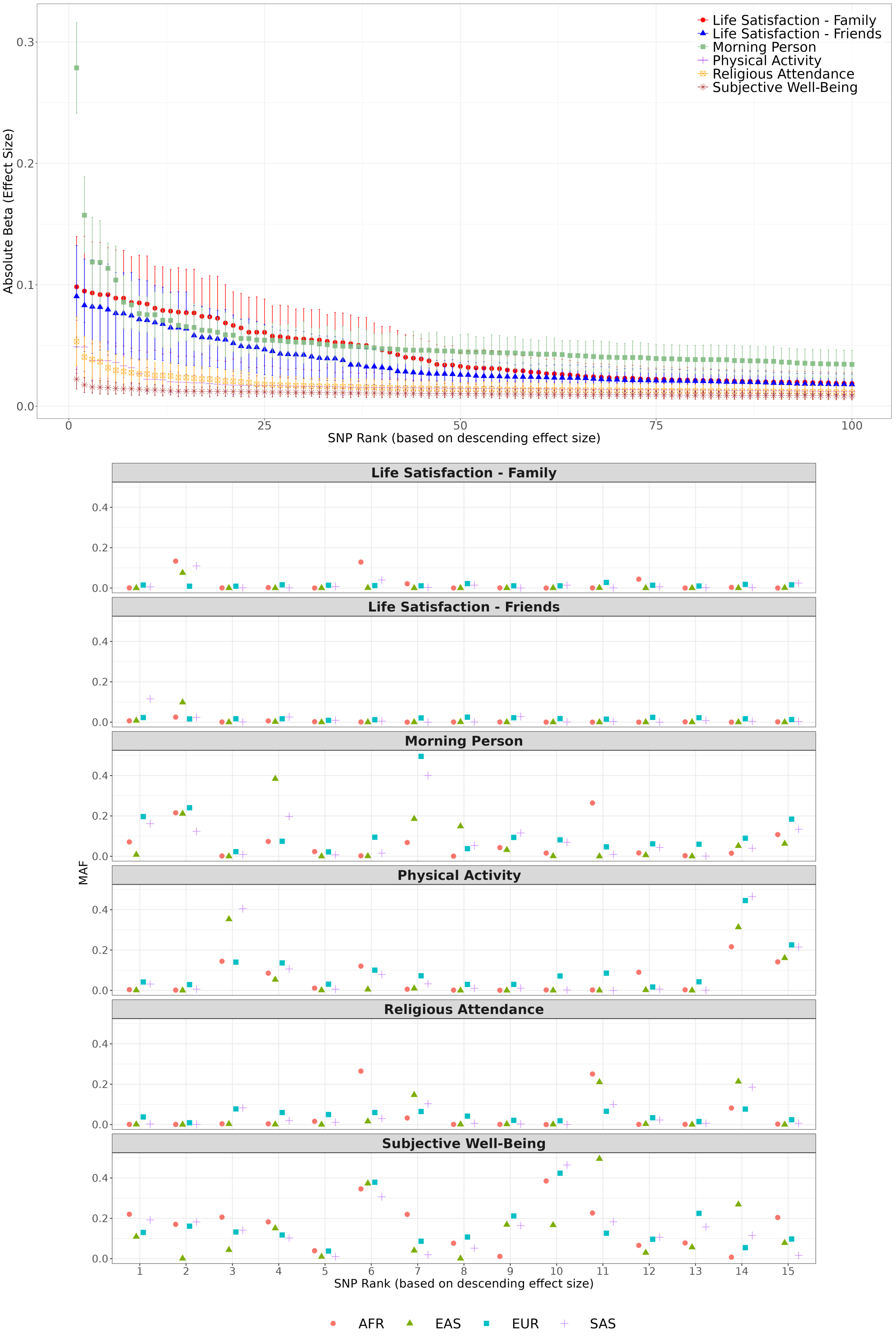

Fig. S14: Effect sizes and minor allele frequencies of SNPs associated with wellbeing and lifestyle traits. The top 100 SNPs were selected based on their p-values and are ordered by descending absolute effect size. **Top panel:** Absolute effect sizes of these top 100 SNPs, ranked accordingly. **Bottom panel:** Minor allele frequencies (MAF) for the top 15 large-effect SNPs disaggregated by genetic ancestry (EUR, SAS, AFR, and EAS) as estimated from the 1KGP reference panel[38]. For both panels, MAF is computed as the minimum of the reported alternate allele frequency and its complement (i.e. $\text{MAF} = \min(\text{ALT\_FREQ}, 1 - \text{ALT\_FREQ})$).

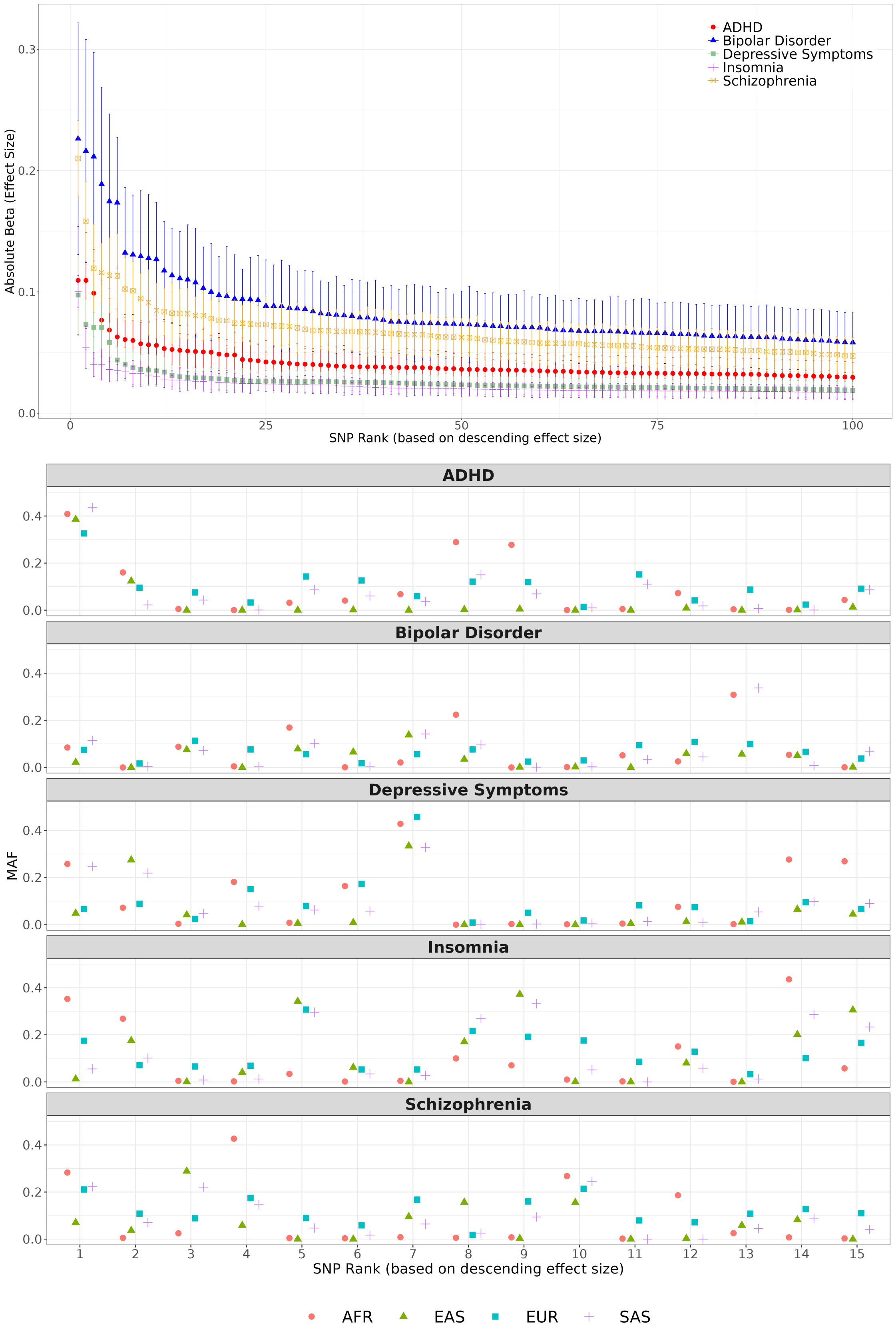

Fig. S15: Effect sizes and minor allele frequencies of SNPs associated with psychiatric conditions. The top 100 SNPs were selected based on their p-values and are ordered by descending absolute effect size. **Top panel:** Absolute effect sizes of these top 100 SNPs, ranked accordingly. **Bottom panel:** Minor allele frequencies (MAF) for the top 15 large-effect SNPs disaggregated by genetic ancestry (EUR, SAS, AFR, and EAS) as estimated from the 1KGP reference panel[38]. For both panels, MAF is computed as the minimum of the reported alternate allele frequency and its complement (i.e. $\text{MAF} = \min(\text{ALT\_FREQ}, 1 - \text{ALT\_FREQ})$).

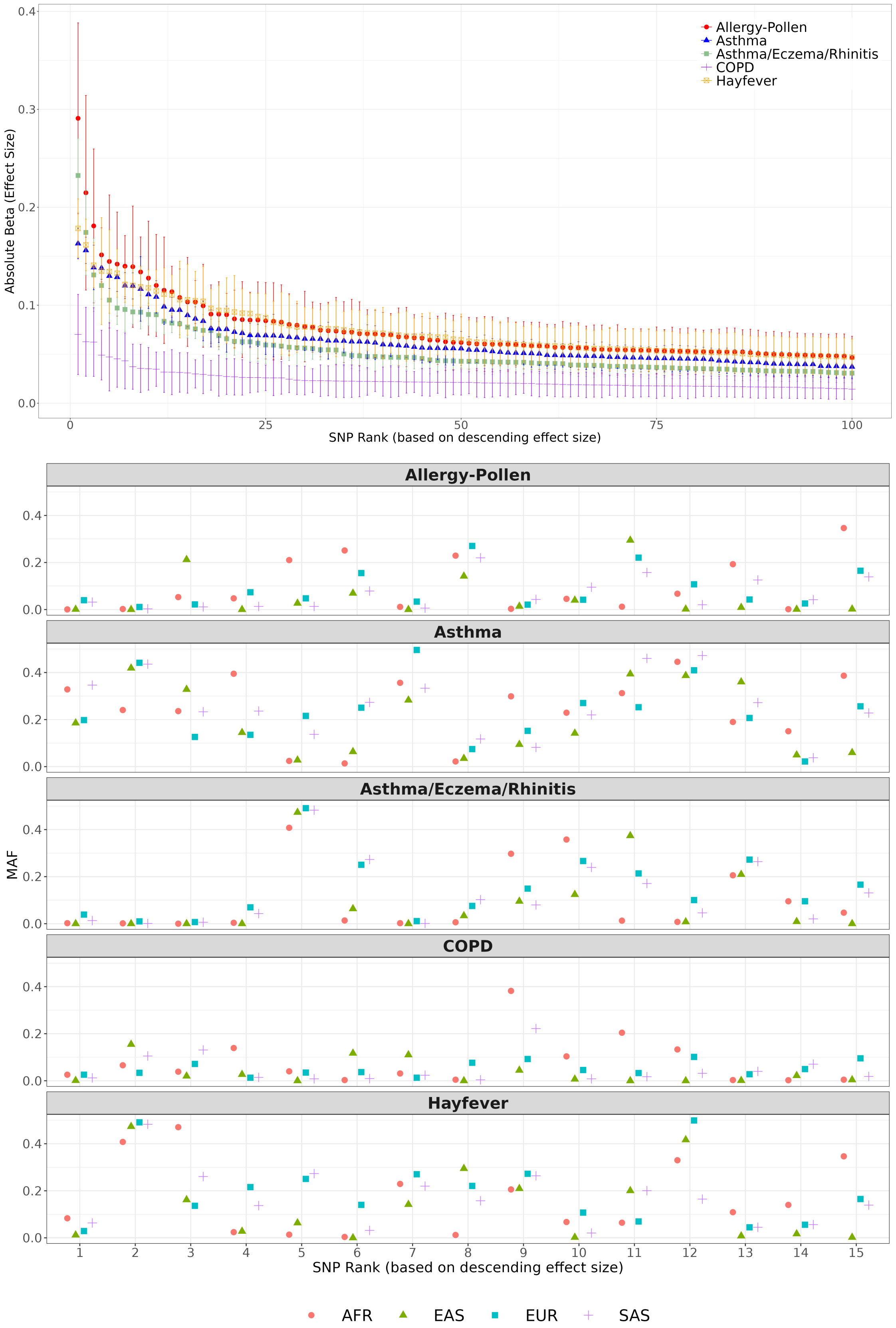

Fig. S16: Effect sizes and minor allele frequencies of SNPs associated with respiratory and allergic conditions. The top 100 SNPs were selected based on their p-values and are ordered by descending absolute effect size. **Top panel:** Absolute effect sizes of these top 100 SNPs, ranked accordingly. **Bottom panel:** Minor allele frequencies (MAF) for the top 15 large-effect SNPs disaggregated by genetic ancestry (EUR, SAS, AFR, and EAS) as estimated from the 1KGP reference panel[38]. For both panels, MAF is computed as the minimum of the reported alternate allele frequency and its complement (i.e. $\text{MAF} = \min(\text{ALT\_FREQ}, 1 - \text{ALT\_FREQ})$).

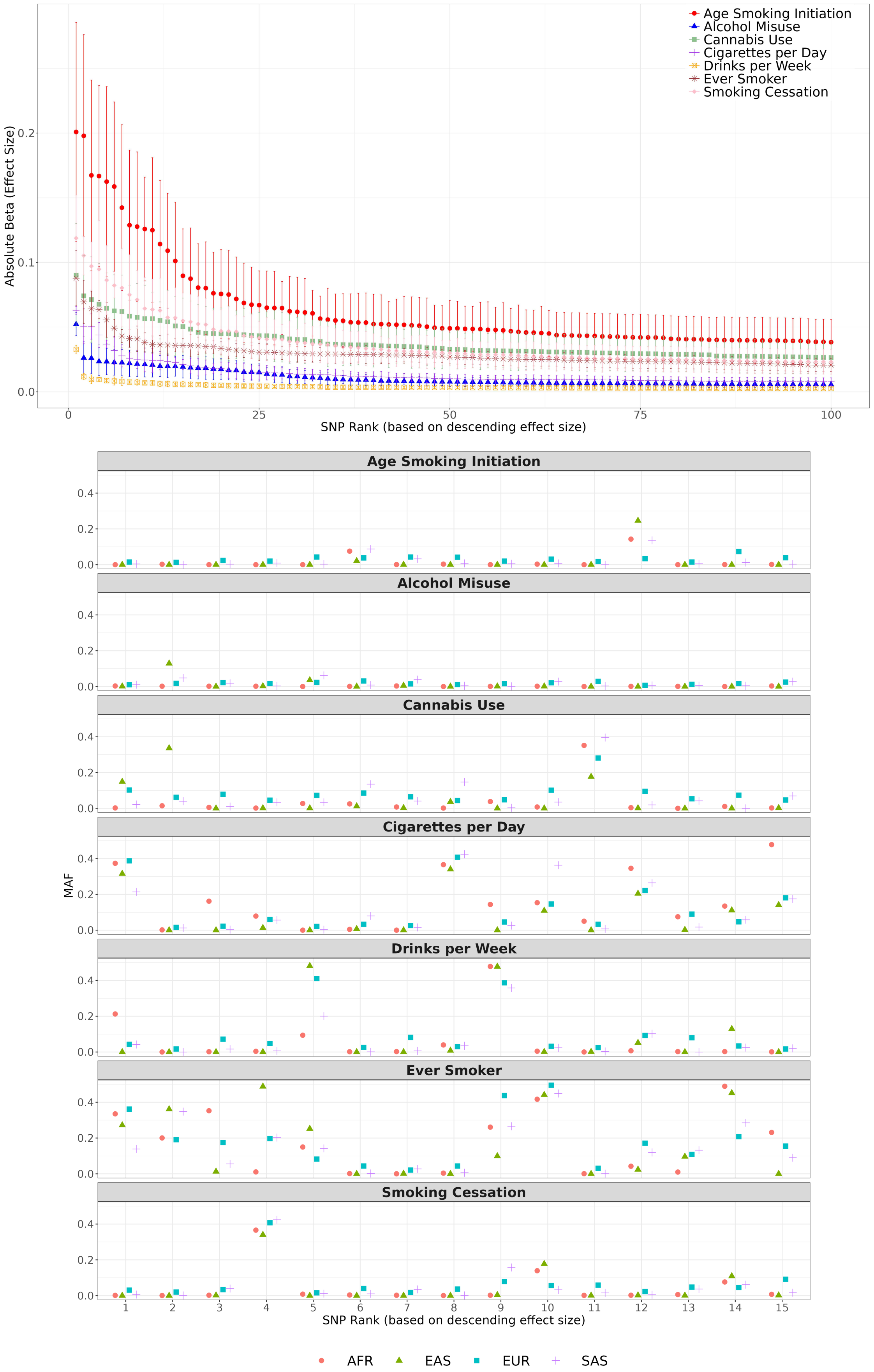

Fig. S17: Effect sizes and minor allele frequencies of the top 100 SNPs associated with substance use traits. The top 100 SNPs were selected based on their p-values and are ordered by descending absolute effect size. **Top panel:** Absolute effect sizes of these top 100 SNPs, ranked accordingly. **Bottom panel:** Minor allele frequencies (MAF) for the top 15 large-effect SNPs disaggregated by genetic ancestry (EUR, SAS, AFR, and EAS) as estimated from the 1KGP reference panel[38]. For both panels, MAF is computed as the minimum of the reported alternate allele frequency and its complement (i.e. $\text{MAF} = \min(\text{ALT\_FREQ}, 1 - \text{ALT\_FREQ})$).
